# Effects of Different Forage Sources on Growth Performance, Blood Biochemistry, Hormone Concentrations, and Intestinal Microbiota in Alpacas

**DOI:** 10.3390/ani15172625

**Published:** 2025-09-08

**Authors:** Zhihui Chen, Yang Zhao, Liangmei Xu, Teng Teng, Deying Ma

**Affiliations:** College of Animal Science and Technology, Northeast Agricultural University, Harbin 150030, China

**Keywords:** alfalfa, alpacas, corn straw, gut microbiota, *Leymus chinensis*, oat grass

## Abstract

Alpaca farming has received increasing attention in China in recent years. However, Australian alfalfa remains the main choice of forage for alpacas. China has abundant forage resources, including oat grass, *Leymus chinensis*, and corn straw, which are widely distributed across the country. This study aims to explore the feasibility of replacing Australian alfalfa entirely with these resources, thereby providing insights into the selection of forage species for alpacas. Test results showed that oat grass, when used as a fiber source for alpacas, has a significant positive effect on their growth performance, energy metabolism and intestinal microbiota. These results suggest that oat grass is a viable forage option for alpacas and is as nutritionally adequate as alfalfa. In contrast, neither corn straw nor *Leymus chinensis* demonstrated superior nutritional value compared to alfalfa. Therefore, we conclude that oat grass has substantial potential to serve as an alfalfa substitute in alpaca forage. Conversely, *Leymus chinensis* and corn straw are not suitable candidates for replacing alfalfa.

## Simple Summary

The alpaca is an emerging special economic animal that has seen a steady increase in the scale of farming in China. However, research into alpacas’ forage development and alpacas’ nutritional requirements remains incomplete. Australian alfalfa remains the primary forage used to feed alpacas, while research into utilizing other types of forage is still limited. Oat grass, *Leymus chinensis*, and corn straw are abundant native forage resources in China with high yields and favorable feeding value. This study therefore aims to assess the suitability of these three forages as alfalfa substitutes in alpaca diets, and to systematically investigate their impact on growth performance, blood biochemical indices, hormonal profiles, and intestinal microbiota composition.

## 1. Introduction

Since the first introduction of alpacas to China in 2002, China has developed a breeding history spanning over two decades [1]. Today, alpacas have become a highly valued animal in the Chinese livestock industry, prized for their high-quality wool, milk, and meat, among other resources [2]. Their gentle temperament and endearing appearance have also made them popular as companion animals. In recent times, a number of Asian countries, including China, have established significant alpaca farming industries. Alpaca farming primarily uses alfalfa, with Australian alfalfa being favored. By 2020, China’s imports of alfalfa hay had risen from 440,000 tonnes to 1.36 million tonnes, while the self-sufficiency rate for high-quality alfalfa stood at just 64% [3]. Due to low domestic alfalfa production capacity, China is highly dependent on imports. Moreover, there are quality differences between Chinese and Australian alfalfa due to regional and climatic variations [4,5]. Therefore, exploring the use of alternative forage species in alpaca feed is highly significant. 

*Leymus chinensis*, a dominant forage in northern Chinese grasslands, is rich in crude fat, crude fiber, and crude protein. It features a high yield, salt-alkali tolerance and good palatability [6,7]. Oat grass, which is native to China, is an annual herb renowned for its high yield, strong stress resistance, and soil-improving properties [8,9]. Rich in water-soluble carbohydrates and highly palatable, it has become a premium source of forage for herbivores [10]. Corn straw, the residual stems and leaves left over from harvesting corn, is a highly abundant resource containing a variety of carbohydrates and has been widely used in feeding ruminants in recent years [9]. Alfalfa, the traditional fodder used in alpaca farming, contains essential amino acids, minerals, vitamins and dietary fiber [11]. Its polyphenolic compounds give it antioxidant properties, and the UN Food and Agriculture Organization recognizes it as an exceptional source of high-quality protein [12,13]. Oat grass, *Leymus chinensis*, and corn straw are three high-yielding, native Chinese forages that could potentially be used as an alternative to alfalfa. Their impact on alpaca health and nutrition should be evaluated.

So far, research on alpaca nutritional requirements and forage selection in China has primarily relied on findings from South American studies or extrapolations from other ruminants, such as cattle and sheep. However, given the significant differences in alpacas’ digestive systems compared to other ruminants, as well as the regional variability in their nutritional needs, previous assessment models have proven outdated and inaccurate [14,15]. Given the smaller rumen volume of alpacas compared to conventional ruminants such as cattle and sheep, and the absence of an omasum, alpacas retain chyme in the rumen for a notably shorter time [16]. Consequently, for digestion, alpacas rely more heavily on hindgut fermentation in the cecum and colon, compared to cattle and sheep [17]. Gut microbiota are key contributors to hindgut fermentation and play a pivotal role in this process [18,19]. The assessment of how different forage species affect the microbial communities in the hindgut of alpacas is also significant in the context of evaluating the nutritional value of forage. Therefore, this study focuses on characterizing the composition and functional traits of alpaca intestinal microbiota under various forage conditions, and evaluating how these microbial communities adapt to and utilize different types of forage.

This study investigated the differences in the growth performance, blood biochemical indices, blood hormones, and intestinal microbiota of alpacas fed four different forage sources: oat grass, *Leymus chinensis*, stover, and alfalfa. It is hypothesized that oat grass, sheep grass and straw—three types of native Chinese forage grass—have the potential to replace alfalfa as a feed source for alpacas without negatively impacting their health or hindgut microbial fermentation capacity.

## 2. Methods

### 2.1. Animals and Experimental Design

In this study, twelve two-year-old female alpacas were randomly assigned to four groups based on different feed fiber sources: alfalfa (MX, *n* = 3), oat grass (YMC, *n* = 3), *Leymus chinensis* (YC, *n* = 3), and corn straw (JG, *n* = 3). Each group consisted of three replicates, with one alpaca per replicate. There were no significant differences in weight among the alpacas across the groups (initial body weight = 52.75 ± 2.96 kg). Alpacas were fed twice daily at 8 a.m. and 4 p.m. All steers were given a commercial total mixed ration (TMR). The composition of the TMR and the nutritional levels for each group are shown in Table 1. All alpacas were provided with free access to feed and water, and manure was removed once daily. Weekly in vivo and in vitro deworming of the subject animals was conducted. The experimental period spanned 60 days. The enclosures comprised a double-layer fence per each alpaca, with an inner core area of 16 square meters (4 m × 4 m), an outer perimeter width of 2 m, an inner height of 1.5 m, and an outer height of 1 m. The laboratory animals and facilities were provided by the Harbin Veterinary Research Institute and were housed at the Animal Testing Base of the Harbin Veterinary Research Institute in Harbin City, Heilongjiang Province.

### 2.2. Sample Collections

From days 57 to 59 of the experiment, alpacas’ feces were collected according to published procedures [20]. Twice a day, at 07:00 and 20:00, individual alpacas’ feces were collected and weighed. A daily total of 1 kg of feces was obtained from each alpaca and placed in a clean stainless-steel tray. The feces were mix thoroughly three times with a shovel to eliminate clumps or layers. The tray was then divided into four equal sections with a ruler; the two diagonal samples were discarded, and the remaining two were remixed. This spreading, dividing, and discarding process was repeated until the sample was reduced to 200 g. This amount of 200 g was sampled, with 100 g mixed with 10 g of 10% hydrochloric acid and dried at 60 °C for 72 h. The dried samples were then crushed, sieved through a 40-mesh sieve, and bagged for testing. All feed samples were crushed, sieved through a 40-mesh sieve, and stored in bags. Additionally, 20 g of fresh alpaca feces was collected, placed in sterilized and disinfected cryogenic tubes, and stored at −80 °C. Another 10 g of fecal samples was used for 16S rRNA detection. On day 60 of the experiment, blood was collected from the alpacas using blood collection tubes. The alpacas were fasted for 12 h before collection. Twenty milliliters of whole blood were collected from each alpaca. The collected whole blood was centrifuged at 3500 r/min for 10 min, and the serum was separated using a pipette and stored at −20 °C. 

### 2.3. Growth Performance and Nutrient Digestibility

All alpacas were weighed at the beginning and at the end of the trial. The amount of feed taken by the alpacas was recorded daily. The diets were analyzed for dry matter (DM) by GB/T 6438-2022 [21], crude protein (CP) GB/T 6432-2018 [22], crude fiber (CF) GB/T 6434-2022 [23], calcium (Ca) GB/T 6436-2018 [24] and phosphorus (P) GB/T 6437-2018 [25]. Nutrient levels were determined according to Chinese national standards, and nutrient digestibility was calculated for each nutrient. The dry matter intake of roughage and concentrate feed during the last three days of the trial period was recorded. The total nutrient intake based on the nutrient content of the roughage and concentrate feed was calculated. The nutrient content of the feces and feeds was determined.

$$Apparent\;nutrient\;digestibility\;(\%)=\frac{Total\;intake\;of\;the\;nutrient-Total\;excretion\;of\;the\;nutrient}{Total\;intake\;of\;the\;nutrient}\times 100\%$$

### 2.4. Plasma Biochemical Analysis, Hormones, and Antioxidant Enzymes Activity

Glucose (GLU), urea nitrogen (BUN), triglycerides (TG), total protein (TP), total bilirubin (TBIL), alanine aminotransferase (ALT), aspartate aminotransferase (AST) and lactate dehydrogenase (LDH) concentrations in the alpacas’ serum were measured using the Switzerland Deakin Tecan Genios multifunctional enzyme labeling instrument. Serum glutathione peroxidase (GSH-Px), malondialdehyde (MDA), total superoxide dismutase (T-SOD), and total antioxidant capacity (T-AOC) were measured by enzyme-linked immunosorbent assay (ELISA) kits from Jiangsu Jingmei biotechnology company. Serum concentrations of growth hormone (GH), insulin (INS), angiotensin II (ANGII), Adrenocorticotropic Hormone (ACTH), and norepinephrine (NE) were measured by enzyme-linked immunosorbent assay (ELISA) kits from the Nanjing Jiancheng biotechnology company. The catalog numbers of ELISA kits are shown in Supplementary Table S1. Measurements were performed at the corresponding absorbance using an enzyme marker (Infinite 200 pro; TECAN) according to the instructions.

### 2.5. 16S rDNA Gene Sequencing Analysis

The QIAamp DNA Stool Mini kit (Qiagen, Hilden, Germany) was used to extract the total bacterial DNA from fresh feces (*n* = 3). An enzyme marker (Infinite 200 pro; TECAN, Menendorf, Switzerland) was used to measure the OD value of total DNA. The quality of the total DNA was determined by confirming that the ratio of OD260 and OD280 was between 1.8 and 2.0. The V4 hypervariable region of the 16S rRNA gene was PCR-amplified using the primers 515F and 806R, which harbored Illumina adaptors and molecular barcodes. Next, paired-end sequencing was carried out on the Illumina HiSeq 2500 platform (Bioacme Coa, Wuhan, China). Raw reads in this study were filtered and merged as raw tags through the FASTP. Raw tags were filtered to produce clean tags. After that, clean tags were used for clustering to obtain operational taxonomic units after the quality filter. Then, the abundance of operational taxonomic units was calculated.

### 2.6. Statistical Analysis 

Firstly, the data in this study were integrated and calculated using Microsoft Excel 2025. Each alpaca was considered the experimental unit. Then, we evaluated the normality and homogeneity of variances in the data in this research in SPSS 28.0 (IBM-SPSS Inc., Chicago, IL, USA). Next, the significance of differences was analyzed by “*t*-test” in SPSS 28.0 (IBM-SPSS Inc., Chicago, IL, USA). The statistical analysis of intestinal microbiota was performed using the “Tukey–Kramer” method. The data were visualized by GraphPad Prism (version 8.0.2, GraphPad Software Inc., SanDiego, CA, USA). Data in this research are expressed as mean ± SEM. Differences were considered significant when *p* < 0.05. Additionally, “*” means *p* < 0.05, and “**” means *p* < 0.01. The programming language R (version 4.2.1, The Comprehensive R Archive Network, USA) was used for correlation analysis involved in this research.

## 3. Results

### 3.1. Effects of Different Fiber Sources on Growth Performance and Nutrient Digestibility of Alpacas

The effects of different fiber sources on the growth performance of alpacas are shown in Table 2. Compared with MX, the average daily feed intake of the YC (*p* = 0.003) and JG (*p* = 0.002) groups was significantly lower. Compared with the YMC group, the average daily feed intake of the YC (*p* = 0.002) and JG (*p* = 0.001) groups was also significantly lower. Still, no significant difference was observed between the YMC and MX groups (*p* = 0.991). The average daily gain of alpacas in the YMC group was significantly greater than that observed in the YC (*p* = 0.048) and JG groups (*p* = 0.004). Furthermore, the inclusion of corn straw and *Leymus chinensis* did not influence the average daily gain of the alpacas (*p* = 0.297). Moreover, the F:G of the YMC group was significantly lower than that of the JG group (*p* = 0.008). In comparison to the MX group, there was no significant difference in the F:G among the YMC (*p* = 0.329), YC (*p* = 0.997), and JG (*p* = 0.102) groups. 

### 3.2. Effects of Different Fiber Sources on Nutrient Digestibility of Alpacas

**Figure 1:**
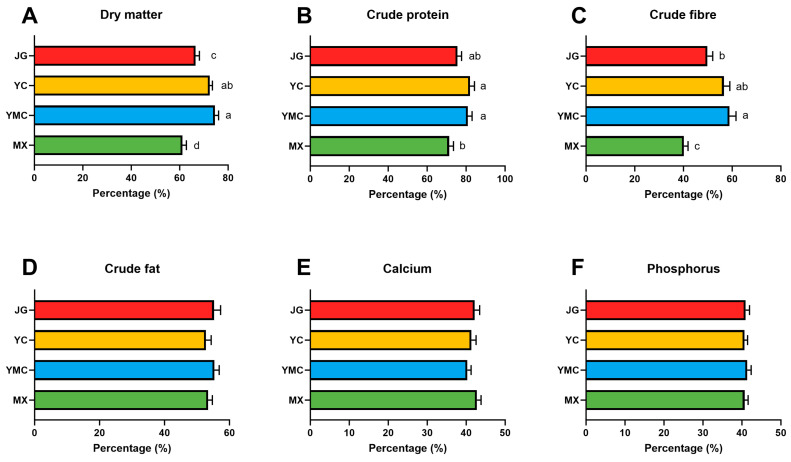
Effects of different fiber sources on the nutrient digestion level of alpacas. (**A**–**F**) Nutrient utilization rates of dry matter, crude protein, crude fiber, crude fat, calcium, and phosphorus. Data were displayed as the means ± standard error of measurement (SEM); Different colours represent different groups. a–d means within a row with no common superscripts difference (*p* < 0.05).

We assessed the effects of different fiber sources on the Apparent Total Tract Digestibility (ATTD) of nutrients in alpacas. Utilizing oat grass, *Leymus chinensis*, and corn straw as fiber sources enhanced the ATTD of dietary nutrient dry matter in alpacas (Figure 1A, *p* = 0.005). The dry matter ATTD of oat grass was significantly higher than that of corn straw (*p* = 0.005), while no significant difference was found when compared to the YC group (*p* = 0.343). Moreover, the ATTD of crude protein in the MX group was significantly lower than that observed in the YMC (*p* = 0.015) and YC (*p* = 0.009) groups (Figure 1B), although no significant difference was detected between other groups. In addition, the crude fiber ATTD in the MX group was significantly lower than that in the YMC (*p* < 0.001), YC (*p* < 0.001), and JG (*p* = 0.017) groups. The crude fiber ATTD in the YMC group was significantly higher than that in the JG (*p* = 0.023) group, and no significant differences were detected between the other groups (Figure 1C). In addition, no changes were detected in the ATTD of crude fat (*p* = 0.599), calcium (*p* = 0.484), and phosphorus (*p* = 0.942) in the other three groups compared with the MX group (Figure 1D–F).

### 3.3. Effects of Different Fiber Sources on Blood Biochemical Parameters of Alpacas

Table 3 illustrates the impact of various fiber sources on the biochemical parameters of the plasma in alpacas. Utilizing oat grass, *Leymus chinensis*, and corn straw as sources of fiber did not significantly influence the concentration of blood urea nitrogen (BUN) (*p* = 0.99), glucose (GLU) (*p* = 0.94), total protein (TP) (*p* = 0.63), alanine aminotransferase (ALT) (*p* = 0.099), and total bilirubin (TBIL) (*p* = 0.398) in alpacas’ plasma. The TG concentration in the YMC group was significantly elevated compared to the MX (*p* = 0.006), YC (*p* < 0.001), and JG (*p* = 0.001) groups. The AST concentration in the JG group exhibited a tendency to be higher compared to the other three groups (*p* = 0.072). Moreover, the LDH concentration was significantly higher in the JG group than in the MX (*p* < 0.001), YMC (*p* < 0.001), and YC (*p* < 0.001) groups. 

### 3.4. Effects of Different Fiber Sources on Serum Antioxidant Function of Alpacas

Next, we examined the effects of different fiber sources on the antioxidant capacity of alpacas’ serum (Table 4). The results indicated that the serum glutathione peroxidase (GSH-Px) activity in the JG group was significantly higher than that in the MX (*p* = 0.017) and YC groups (*p* = 0.021). The serum GSH-Px activity in the YMC group had no significant changes compared with the other three groups (*p* > 0.05). Furthermore, relative to the MX group, no significant alterations in serum GSH-Px activity were observed in the YMC, YC, and JG groups.

### 3.5. Effects of Different Fiber Sources on Serum Endocrine Hormone Concentrations in Alpacas

Subsequently, we examined the impact of various fiber sources on serum endocrine hormone concentrations in alpacas (Table 5). The administration of corn straw to alpacas resulted in a significant elevation of serum angiotensin II (ANGII) concentrations (*p* = 0.007). Compared with the MX group, feeding alpacas with oat grass, *Leymus chinensis*, and corn straw had no effect on serum adrenocorticotropin (ACTH), norepinephrine (NE), and insulin (INS) concentrations (*p* > 0.05). Notably, the serum growth hormone (GH) concentration was significantly elevated in the YMC group relative to the MX (*p* = 0.018), YC (*p* = 0.043), and JG (*p* < 0.001) groups. Furthermore, the serum GH concentration in the JG group was significantly reduced compared to the MX group (*p* = 0.003).

### 3.6. Effects of Different Fiber Sources on Alpha Fecal Microbial Diversity and Phylum-Level Composition

**Figure 2:**
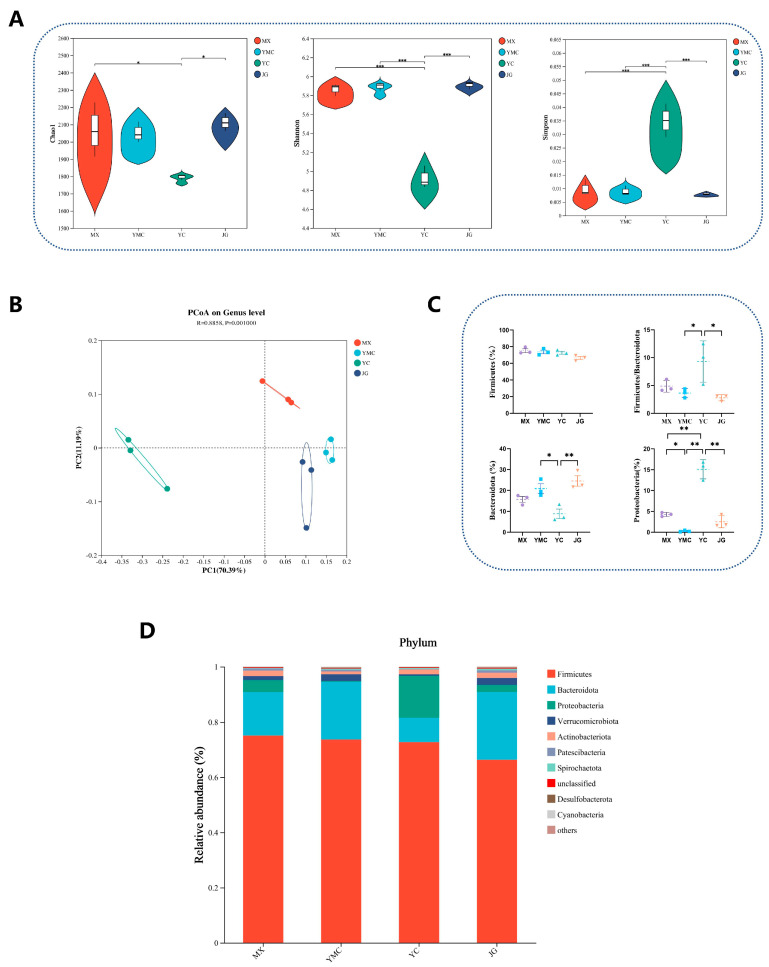
Effect of different fiber sources on alpha fecal microbial diversity and phylum-level composition. (**A**) Chao1, Shannon and Sipson indices of the fecal microbial composition. (**B**) PCoA analysis of the microbial compositional profiles. (**C**) Bacterial composition at the phylum level of colonic microbiota. (**D**) Firmicute, Bacteroidete, and Verrucumicrobiota levels of fecal microbiota. Data were displayed as the means ± standard error of measurement (SEM); Different colours represent different groups. “*” means *p* < 0.05, “**” means *p* < 0.01, “***” means *p* < 0.001.

We evaluated the impact of different dietary fiber sources on the microbial composition of alpacas’ feces. We first observed the alpha diversity index of the colonic microbiota (Figure 2A). Compared with the other three fiber sources, supplementation with *Leymus chinensis* as a fiber source significantly decreased the Shannon diversity index (Figure 2A, *p* < 0.001) while increasing the Simpson index of the fecal microbiota (Figure 2A, *p* < 0.001). In addition, the YC group exhibited a significantly lower Chao1 richness index than the MX (*p* < 0.001) and JG (*p* < 0.001) groups, whereas no significant difference was observed when compared with the YMC group (Figure 2A). The alpha diversity of fecal microbiota in cold-exposed alpacas was altered by dietary fiber sources. The PCoA analysis showed that different fiber sources significantly changed the microbiota (Figure 2B, *p* = 0.001). Subsequently, we investigated the effects of various fiber sources on the relative abundance of dominant microbial phyla in alpacas’ feces. The predominant phyla identified across the four experimental groups included Firmicutes, Bacteroidetes, Proteobacteria, Verrucomicrobia, and Actinobacteria (Figure 2C). Notably, the relative abundance of Proteobacteria in the fecal microbiota was significantly higher in the YC group compared to the MX (*p* < 0.001), YMC (*p* < 0.001) and JG (*p* < 0.001) groups (Figure 2D). 

### 3.7. Effects of Different Fiber Sources on the Alpha Fecal Microbial Genus Level

**Figure 3:**
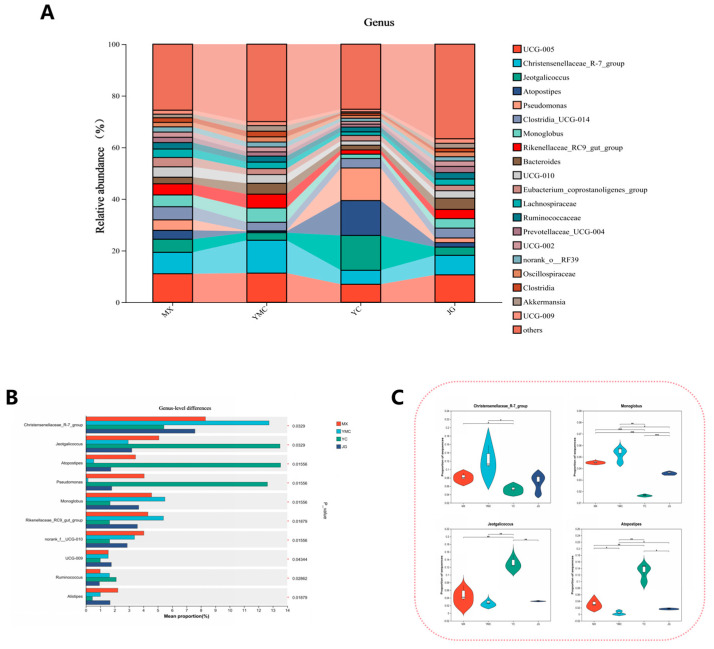
Effect of different fiber sources on the alpha fecal microbial genus level. (**A**) Bacterial composition at the genus level of fecal microbiota. (**B**) Genera with differences in abundance among the top 30 richest genera. (**C**) Several genera with significant differences in abundance are outside the top 30 richest genera. Data were displayed as the means ± standard error of measurement (SEM). Different colours represent different groups. “*” means *p* < 0.05, “**” means *p* < 0.01, “***” means *p* < 0.001.

The predominant genera identified across the four experimental groups included UCG-005, *Christensenellaceae* R-7 group, *Jeotgalicocus*, *Atopostipes*, and *Pseudomonas* (Figure 3A). Furthermore, different fiber sources significantly reshaped the composition of the alpacas’ fecal microbiota at the genus level. The relative abundances of *Christensenellaceae* R-7 group, *Jeotgalicoccus*, *Atopostipes*, and *Pseudomonas* varied notably among the dietary treatments (Figure 3B). Specifically, the abundance of *Christensenellaceae* R-7 group was significantly lower in the YC group compared to the MX (*p* = 0.036) and YMC (*p* = 0.030) groups (Figure 3C), indicating a fiber source-dependent reduction. Conversely, the YC group exhibited a significantly higher abundance of *Jeotgalicoccus* relative to the other groups (Figure 3C, *p* = 0.033). For the genus *Monglobus*, significant differences were observed among groups (*p* = 0.016), except between the MX and YMC groups (*p* = 0.071), which showed no statistical difference (Figure 3C). Similarly, *Atopostipes* displayed significant variation across all groups (Figure 3C, *p* = 0.016), with the exception of the MX and JG groups (*p* = 0.064), where no significant difference was detected (Figure 3C).

### 3.8. Effects of Different Fiber Sources on Alpha Fecal Microbial Marker Bacteria and Their Functions

**Figure 4:**
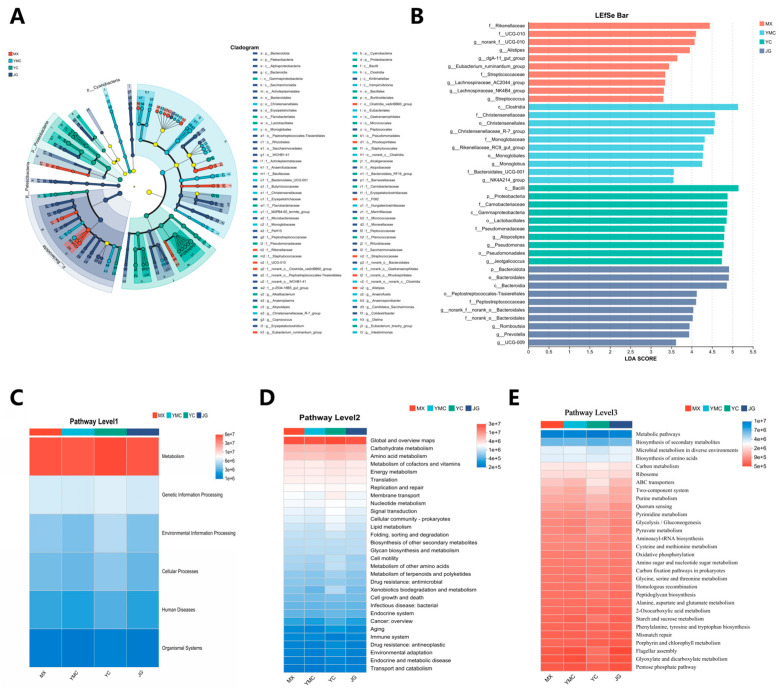
Effects of different fiber sources on the fecal microbial communities of alpacas. (**A**,**B**) LEfSe result of differences in microbial abundance. (**C**–**E**) KEGG analysis of fecal microbiota of alphas.

The linear discriminant analysis (LDA) effect size (LEfSe) was used to identify differences in the gut microbiota among three groups, and an LDA score ≥3.5 was displayed (Figure 4A). Our results showed that the YMC group was enriched in members of the *Christensenellaceae* R-7 group, the *Rikenellaceae RC9 gut group*, *Monoglobus*, and the *NK4A214 group*. In contrast, the YC group exhibited higher relative abundances of *Atopostipes*, *Pseudomonas*, and *Jeotgalicoccus*. Additionally, the JG group was characterized by greater enrichment of *UCG-009*, *Prevotella*, and *Romboutsia* (Figure 4A). KEGG revealed that the different fiber resources were abundant in metabolism (Figure 4B). In KEGG pathway level2, carbohydrate metabolism and amino acid metabolism were abundant (Figure 4D), and in KEGG pathway level3, pentose phosphate pathway was also abundant (Figure 4E). 

### 3.9. Effects of Different Fiber Sources on Fecal Microbial Composition in Alpha

**Figure 5:**
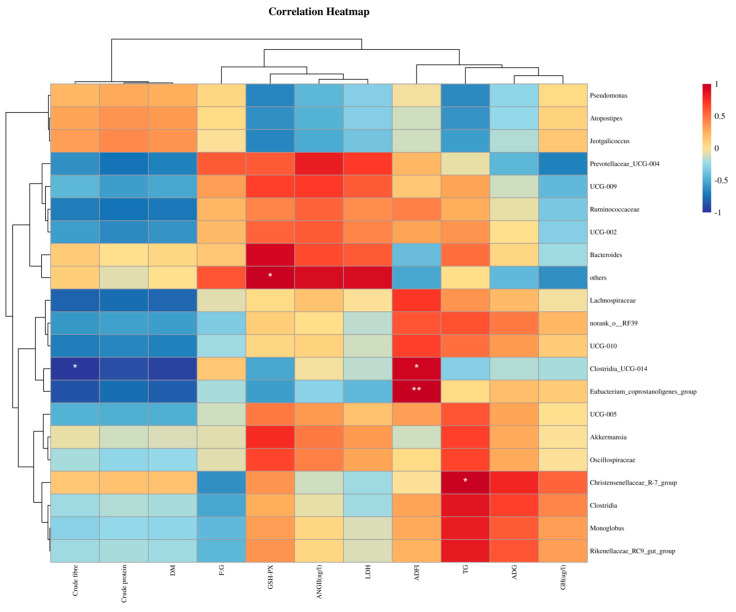
Spearman correlation between growth performance, blood biochemistry, blood antioxidants, blood hormone concentrations and relative abundance of microbial genera. The R-values are shown in different colors, with red and blue indicating positive and negative correlation, respectively. * *p* < 0.05, **p < 0.01 compared to the control group. Data were displayed as the means ± standard error of measurement (SEM); “*” means *p* < 0.05, “**” means *p* < 0.01.

We further explored the relationships between fora and Growth performance, blood biochemistry, and blood hormone concentrations via Spearman correlation analysis (Figure 5). The *Eubacterium coprostanoligenes* group was positively correlated with ADFI. *Clostridia* UCG-014 was significantly positively correlated with ADFI and significantly negatively correlated with crude fiber digestibility. The *Christensenellaceae* R-7 group was significantly positively correlated with TG (Figure 5).

## 4. Discussion

Alpacas exhibit distinct feeding preferences due to the varying palatability of different forage species. Alfalfa is widely acknowledged as a forage with excellent palatability [26,27]. The palatability of *Leymus chinensis*, oat grass, and corn straw for alpacas, along with the effects of different forage types on their growth and development, are fundamental to determining whether these local forages can be utilized. In this study, it was demonstrated that oat grass also exhibited a significantly high feeding value for alpacas. The utilization of alfalfa and oat grass as fiber sources resulted in a substantial increase in feed intake among alpacas in comparison to the use of *Leymus chinensis* and corn straw. However, only oat grass significantly improved the average daily gain (ADG) of alpacas when compared to *Leymus chinensis* and corn straw, indicating that oat grass is highly palatable and that alpacas exhibit strong nutritional utilization capacity for oat grass. As anticipated, the oat straw group demonstrated the lowest feed conversion ratio (F:G) among the four forage groups. While only the corn stover group demonstrated a statistically significant difference compared to the oat straw group, this further corroborates the high economic value of oat straw as alpaca feed. Oat grass has been demonstrated to enhance feed digestibility in ruminants [28] and to improve rumen fermentation patterns and nitrogen utilization efficiency [29]. Consequently, an evaluation was conducted to ascertain the apparent digestibility of DM, CP, CF, EE, Ca, and P for the four forages. The apparent digestibility of DM, CP, and CF in alpacas fed with oat grass and *Leymus chinensis* was found to be significantly higher than that in the alfalfa and corn straw groups. However, no significant difference was observed between the oat grass and *Leymus chinensis* groups. This finding suggests that the utilization efficiency of oat grass by alpacas exceeds that of alfalfa, thereby underscoring the potential of oat grass to supersede alfalfa as the primary forage source. It is noteworthy that *Leymus chinensis* exhibited high apparent digestibility, which may provide a rationale for the observed lack of significant difference in daily gain between the *Leymus chinensis* and alfalfa groups, despite a substantial reduction in feed intake. This finding indicates that *Leymus chinensis* possesses the capacity to substitute for alfalfa as a forage source, although its overall utilization efficiency is comparatively lower than that of oat grass.

Additionally, we characterized the biochemical profiles of alpaca serum further to better evaluate the impact of different forage types on their metabolic levels. The alpacas’ serum TG concentration in the oat grass group was found to be significantly higher than that observed in the other three forages. TG concentrations have been demonstrated to reflect the deposition of lipids within the body, with an increase in TG concentrations indicating lipid accumulation [30,31]. Oat grass is rich in soluble carbohydrates such as starch and fructans, which can be rapidly broken down into monosaccharides by the alpaca’s body. These monosaccharides then provide sufficient precursors for the synthesis of triglycerides (TG) [32,33]. Concurrently, oat grass contains a relatively high proportion of unsaturated fatty acids, which possess favorable digestive and absorptive properties. These fatty acids have the capacity to participate directly in the process of triglyceride assembly [34,35]. The high TG concentration in alpacas of the oat grass group is indicative of enhanced fat utilization in this dietary category. Although oat grass has a higher fat content than the other three types of forage, all of the forage types that we selected are low in fat. We speculate that the main factor affecting alpaca lipid metabolism levels is the difference in fermentation patterns in their rumen and intestines. Our correlation analysis also revealed a strong correlation between the *Christensenellaceae_*R-7 group and the alpaca serum TG concentration. Consequently, the alpacas in the oat grass group exhibited superior average daily gain. The serum LDL concentration of alpacas in the corn straw group was found to be significantly higher than that observed in the other three forages. However, no significant differences were observed in the total TG concentration of alpacas in the corn straw group when compared with the other groups. This finding suggests that corn straw, when used as a fiber source, may enhance the efficiency of lipid transport and metabolism in alpacas. However, it does not appear to have a favorable effect on lipid deposition.

The oxidative stress levels of alpacas under different pasture conditions were assessed by measuring the oxidative stress levels in their serum. It has been established that antinutritional factors present in forages have the capacity to influence the oxidative stress level of animals. This influence is exerted by way of the impact on nutrient absorption and the subsequent induction of inflammatory responses [36]. It has been established that β-glucan is the primary antinutritional factor present in oat grass, while lignin has been identified as the predominant antinutritional factor in *Leymus chinensis* and corn straw. The impact of these antinutritional factors on the nutritional metabolism process and the oxidative stress level of animals has been demonstrated, except for the corn straw group, where the activity of GSH-Px in alpaca serum was found to be significantly higher than that in the *Leymus chinensis* group and alfalfa group. There were no significant differences in GSH-Px, T-SOD, T-AOC and MDA in the serum of alpacas among the other groups [37]. This finding indicates that when corn stover is used as a feed source, it increases the levels of oxidative stress-related factors in alpaca serum, placing the animals in a state of oxidative stress. In order to counteract oxidative damage, the body increases the synthesis or activity of GSH-Px in order to enhance its ability to clear peroxides. A similar phenomenon has been observed in other animal species [38,39]. However, replacing alfalfa with oat grass and *Leymus chinensis* does not promote oxidative stress in alpacas. This finding suggests that using these two forage species as roughage sources for alpacas does not increase their oxidative stress levels, compared to alfalfa.

To further explore the effects of various forage sources on the growth and digestion of alpacas, we analyzed their blood hormone concentrations. Given their high response efficiency in regulating water-salt balance, we evaluated the concentration of angiotensin II (ANGII) in alpaca serum [40]. ANGII is an important blood hormone that regulates blood pressure and water-electrolyte balance in animals [41]. However, continuous high levels of ANG II interfere with the insulin signaling pathway, leading to abnormal phosphorylation of Insulin Receptor Substrate-1 (IRS-1), and affecting the energy utilization efficiency of animals [42,43]. As expected, we found that using corn straw as a forage source for alpacas significantly increased the concentration of ANGII in alpacas’ serum compared with the other three forages. This indicates that corn straw stimulates the thirst center of alpacas, affecting the role of insulin, and leading to nutrient loss and reduced digestion concentration of corn straw [44]. At the same time, the increase in ANGII caused by corn straw leads to oxidative stress in alpacas, which may also explain the reason for the increase in GSH-Px in alpacas’ serum [45]. Growth hormone (GH) promotes protein deposition and bone development in alpacas. Increased GH improves the nutritional metabolism efficiency of alpacas and increases the weight gain level of alpacas [46]. We found that using oat grass as the main forage source for alpacas significantly increased the concentration of growth hormone (GH) in their serum, compared to the other three forages. Alfalfa also considerably increased GH concentration in alpaca serum, compared to the corn straw group. This suggests that oat grass may promote GH secretion in alpaca serum due to its superior amino acid or lipid composition, thereby enhancing the nutritional metabolism level and nutrient deposition capacity of alpacas [47]. An increase in ANG II inhibits GH secretion, thereby limiting muscle and bone development [45]. An increase in ANGII may also be the main reason for the significant downregulation of GH in the corn straw group compared with the alfalfa and oat grass groups. We also measured the secretion concentrations of ACTH, NE, and INS in alpaca serum. There were no significant differences in the hormone concentrations of ACTH, NE, and INS in the serum of alpacas treated with these four forages. In summary, the high ANGII concentration in the corn straw group and the inhibition of GH secretion limit the growth and development of alpacas, further proving that corn straw is not suitable as the main forage type for alpacas. In contrast, oat grass can promote the secretion of GH in alpacas’ serum, thereby playing a positive role in their growth and development.

Given the differences in alpaca utilization capacity for these four forages, we characterized alpaca fecal microbiota to analyze the effects of these forages on intestinal fermentation patterns and microbial composition. First, we measured the α diversity indices of alpaca fecal microbiota of alpacas under these four forage sources and found that only the fecal microbiota of alpacas in the *Leymus chinensis* group showed changes, with the Chao1 and Shannon indices significantly decreasing compared to the other three groups, while the Simpson index increased significantly. This indicates that the decrease in the Chao1 and Shannon indices of the fecal microbiota of alpacas in the *Leymus chinensis* group reflects a reduction in microbial species richness [48]. Conversely, the increase in the Simpson index suggests an increase in the uniformity of the fecal microbiota, suggesting that the abundance of dominant bacterial communities in the fecal microbiota of alpacas in the *Leymus chinensis* treatment group is relatively low [49]. We measured the β-diversity in the fecal microbiota of alpacas under four forage treatments and found significant differences. The analysis of the relative abundance of fecal microbial phyla in alpacas under the four forage treatments showed that Firmicutes, Bacteroidetes, Proteobacteria, and Verrucomicrobia were the four most dominant phyla in the alpaca feces from the three forage sources: alfalfa, *Leymus chinensis*, and corn straw. However, the abundance of Bacteroidetes in the feces of alpacas in the *Leymus chinensis* group was significantly lower than that in the oat grass and corn straw groups, and the Firmicutes/Bacteroidetes ratio was significantly higher. Bacteroidetes are important for animal fiber metabolism and utilization, and the Firmicutes/Bacteroidetes ratio often reflects animal energy utilization. This indicates that the intestinal microbiota of alpacas in the *Leymus chinensis* group had poor fiber utilization ability and significantly lower energy utilization efficiency than the oat grass and corn straw groups [18,50,51]. In contrast to these three groups, the top four dominant phyla in the fecal microbiota of alpacas in the oat grass group were Firmicutes, Bacteroidetes, Proteobacteria, and Actinobacteria. The abundance of Proteobacteria in the feces of alpacas under oat grass treatment was significantly lower than that in the other three groups.

We subsequently measured the composition of the fecal microbiota at the genus level in alpacas under the four forage treatments. The *Christensenellaceae* R-7 group plays a role in degrading complex carbohydrates such as cellulose and hemicellulose [52,53], and its abundance in the feces of alpacas in the YC group was significantly lower than that in the YMC and MX groups. *Monoglobus* can synergistically degrade cellulose in the rumen of ruminants and utilize macromolecular plant polysaccharides such as pectin [54,55]. The abundance of *Monoglobus* in the feces of alpacas in the YC group was significantly lower than that in the YMC, MX, and JG groups. Conversely, the abundance of *Monoglobus* in the feces of alpacas in the MX and YMC groups was also significantly higher than that in the JG group. These changes in abundance indicate that the fiber-degrading ability of the fecal microbiota of alpacas is weaker when *Leymus chinensis* and corn straw are used as forage sources than in the YMC and MX groups. Additionally, *Jeotgalicoccus* is a genus with high salt tolerance [56]. We found that the abundance of *Jeotgalicoccus* in the YC group was significantly higher than that in the other three groups. This indicates that the fecal microbiota of alpacas with YC as the main forage source showed higher salt tolerance than the other three groups. This may be due to the influence of *Leymus chinensis* on the intestinal environment of alpacas. *Atopostipes* is a genus that mainly utilizes polysaccharides that are generally low in abundance in the rumen of ruminants but are enriched in the hindgut [57]. The enrichment of this genus may indicate reduced fiber utilization efficiency. We found that *Atopostipes* abundance in the YC group was significantly higher than that in the other three groups. These differences at the genus level suggest that using *Leymus chinensis* as a forage source for alpacas affects the composition of fecal genera and reduces the ability of alpaca microbiota to degrade fiber.

Additionally, we screened for marker microbes using LEfSe analysis. Since oat grass had a positive impact on the growth and health of the alpacas, our focus was on identifying key microbial markers in the feces of the alpacas in the YMC group. We found that the *Christensenellaceae* R-7 group could serve as an important genus-level marker for the YMC group. As the abundance of *Christensenellaceae* R-7 group in the YMC group was also relatively high, we hypothesize that using oat grass as a forage source for alpacas could lead to an increase in the abundance of *Christensenellaceae* R-7 group in alpaca feces, thereby improving fiber degradation. At the same time, we characterized the metabolic pathways enriched by microorganisms in the KEGG database. The primary metabolic pathways were mainly enriched in the fecal microbiota of alpacas from the four forage sources, while the secondary metabolic pathways were mainly enriched in carbohydrate metabolism. This indicates that microorganisms in the intestinal tract of alpacas are primarily involved in carbohydrate metabolism.

We found that the enrichment degree of ABC transporters in the tertiary metabolic pathway was relatively low in the YC group. ABC transporters play an important role in the process of ATP production in bacterial metabolism [58]. Therefore, the energy generated by the metabolism of intestinal microorganisms in the YC group was relatively low, which may explain the relatively low utilization efficiency of *Leymus chinensis*. Using Spearman’s test, we conducted a correlation analysis between the main differentially abundant microbial genera and the main differential growth performance, blood biochemistry, blood hormones, and blood antioxidant capacity of alpacas. We found that the *Christensenellaceae* R-7 group genus was positively correlated with the triglyceride content in the serum. This genus enhances the triglyceride storage capacity of adipocytes by synthesizing butyric acid to activate the GPR43 receptor [50,59]. The two genera of *Firmicutes*, *Clostridia UCG-014* and *Eubacterium coprostanoligenes*, had a positive correlation with ADFI. This may be due to the difference in the types of fiber in the forage. Oat grass and alfalfa enrich these two genera more effectively in the intestine, and they can both produce short-chain fatty acids by fermenting fiber substances. This affects the metabolic level of alpacas and thus promotes their feed intake [60]. In summary, changes in the intestinal microbiota of alpacas caused by different fiber sources reflect how alpacas digest and utilize forage. Compared with the other three forages, *Leymus chinensis* produces greater differences in the intestinal microbiota of alpacas, and the changes in the microbiota are not conducive to the utilization of nutrients by alpacas. However, oat grass enhances the ability of the intestinal microbiota of alpacas to utilize fiber, which benefits the digestion, absorption and utilization of nutrients. It is worth noting that three alpacas (*n* = 3) per group were selected for this experiment due to the high cost of alpaca rearing. This had a significant impact on the statistical efficiency of the data in this paper. However, we still hope that this study can provide some reference for future research on alpaca forage types. For large animals such as ruminants, three replicates per group were also observed in existing studies [61,62,63]. Additionally, previous studies have confirmed that oat hay and alfalfa pellets have similar apparent digestibility in alpacas. Similar results were obtained in this study, and this indirectly lends credibility to the conclusions of this paper [64]. However, it should be recognized that this is the main limitation of this experiment compared to others in this field. We hope that further large-scale feeding experiments involving alpacas can be carried out in future studies to validate the feasibility of using oat grass as alpaca forage.

## 5. Conclusions

In comparison with alfalfa, the apparent digestibility of dry matter, crude protein, and crude fiber in oat grass is significantly higher for alpacas. When used as alpaca feed, oat grass has been demonstrated to enhance triglyceride levels and optimize the fermentation patterns of the hindgut microbiota. The impact of oat grass as a forage source on growth performance is not significantly different from that of alfalfa. In contrast, the Chinese willowherb and corn stover significantly reduced the average daily gain (ADG) of alpacas in comparison with oat grass, indicating that *Leymus chinensis* and corn stover are less suitable than oat grass as forage for alpacas. However, we identified oat grass as a potential substitute for alfalfa as the primary feed for alpacas.

## Figures and Tables

**Table 1:** Ingredients and nutrient composition of experimental diets (%, dry matter basis).

	MX	YMC	YC	JG
Ingredients				
Alfalfa	50.0%	0.0%	0.0%	0.0%
Oat grass	0.0%	50.0%	0.0%	0.0%
*Leymus chinensis*	0.0%	0.00%	50.00%	0.00%
Corn Straw	0.00%	0.00%	0.00%	50.00%
Corn	35.00%	19.50%	13.50%	26.90%
Wheat bran	10.00%	14.80%	17.00%	2.70%
Soybean meal	0.00%	12.00%	16.00%	16.50%
Calcarea carbonica	0.50%	0.00%	0.00%	0.00%
Premix ^a^	0.50%	0.50%	0.50%	0.50%
Sodium chloride	1.00%	1.00%	1.00%	1.00%
Calcium bicarbonate	3.00%	2.20%	2.00%	2.40%
Nutrient composition ^b^				
Digestible energy (MJ)	10.97	10.92	10.95	10.88
Crude protein	13.21%	13.25%	13.22%	13.22%
Acid detergent fiber	19.39%	20.92%	22.81%	24.58%
Calcium	0.91%	0.95%	0.93%	0.97%
Phosphorus	0.69%	0.67%	0.68%	0.63%

^a^ Each kilogram of premix in the experimental group contains 20 ppm Co, 1600 ppm Cu, 6000 ppm Fe, 8000 ppm Mn, 6000 ppm Zn, 50 ppm I, 60 ppm Se, 1.2000 IU/kg VA, 3300 mg/kg VD, 115 mg/kg VE, 24 mg/kg nicotinamide, and 60 μg/kg biotin. ^b^ Digestible energy was a calculated value, while the others were measured values.

**Table 2:** Effects of different fiber sources on the growth performance of alpacas.

Items	MX	YMC	YC	JG	SEM	*p*-Value
ADFI (g/d)	872.87 ± 16.96 ^a^	878.96 ± 13.8 ^a^	759.66 ± 12.01 ^b^	751.77 ± 16.37 ^b^	19.23	<0.001
ADG (g/d)	88.07 ± 4.60 ^ab^	119.48 ± 2.93 ^a^	79.26 ± 15.76 ^b^	55.56 ± 5.56 ^b^	7.85	0.006
F:G	9.95 ± 0.33 ^ab^	7.36 ± 0.07 ^a^	10.21 ± 1.58 ^ab^	13.76 ± 1.18 ^b^	0.81	0.013

ADFI = average daily food intake; ADG = average daily gain; F:G = feed/gain. Data are presented as mean ± SEM. a–b = within a row with no common superscripts difference (*p* < 0.05).

**Table 3:** Effects of different fiber sources on blood biochemical parameters of alpacas.

Items	MX	YMC	YC	JG	SEM	*p*-Value
BUN (mmol/L)	9.55 ± 0.69	9.58 ± 0.79	9.54 ± 0.60	9.71 ± 0.93	0.32	0.99
GLU (mmol/L)	6.13 ± 0.30	6.42 ± 0.41	6.15 ± 0.36	6.23 ± 0.36	0.16	0.94
TP (g/L)	53.43 ± 1.73	56.4 ± 2.02	53.46 ± 2.71	52.45 ± 2.21	1.04	0.63
TG (mmol/L)	0.24 ± 0.01 ^a^	0.32 ± 0.02 ^b^	0.21 ± 0.01 ^a^	0.22 ± 0.01 ^a^	0.05	<0.001
ALT (IU/L)	18.92 ± 0.65	19.32 ± 0.70	18.96 ± 0.91	21.8 ± 0.92	1.72	0.099
AST (IU/L)	189.95 ± 8.74	185.09 ± 9.86	179.69 ± 8.25	217.04 ± 8.84	5.77	0.072
TBIL (μmol/L)	0.83 ± 0.05	0.84 ± 0.07	0.85 ± 0.06	0.97 ± 0.06	0.11	0.398
LDH (IU/L)	368.63 ± 16.52 ^a^	371.93 ± 17.45 ^a^	377.97 ± 17.19 ^a^	558.03 ± 17.91 ^b^	25.29	<0.001

BUN = Blood urea nitrogen; GLU = Glucose; TP = Total protein; TG = Triglycerides; ALT = Alanine aminotransferase; AST = Aspartate aminotransferase; TBIL = Total Bilirubin; LDH = Lactate Dehydrogenase. Data are presented as mean ± SEM. a–b means within a row with no common superscripts difference (*p* < 0.05).

**Table 4:** Effects of different fiber sources on serum antioxidant capacity of alphas.

Items	MX	YMC	YC	JG	SEM	*p*-Value
GSH-Px (U/mL)	103.7 ± 5.88 ^a^	137 ± 8.00 ^ab^	105.88 ± 9.17 ^a^	155.61 ± 12.52 ^b^	7.65	0.011
T-SOD (U/mL)	108.86 ± 8.68	106.69 ± 11.99	133 ± 12.44	137.26 ± 8.66	6.14	0.159
MDA (U/mL)	2.21 ± 0.13	2.13 ± 0.08	2.3 ± 0.04	2.52 ± 0.17	0.07	0.182
T-AOC (mmol/L)	2.61 ± 0.15	2.57 ± 0.08	2.52 ± 0.21	2.51 ± 0.22	2.39	0.975

GSH-Px = Glutathione peroxidase; T-SOD = Total superoxide dismutase; MDA = Malondialdehyde; T-AOC = Total antioxidative capacity. Data are presented as mean ± SEM. a–b means within a row with no common superscripts difference (*p* < 0.05).

**Table 5:** Effects of different fiber sources on serum hormone concentration of alpacas.

Items	MX	YMC	YC	JG	SEM	*p*-Value
ACTH (ng/L)	41.96 ± 2.63	41.49 ± 1.85	40.18 ± 2.05	45.61 ± 1.67	1.08	0.356
ANGII (ng/L)	24.45 ± 0.32 ^a^	24.2 ± 0.82 ^a^	23.74 ± 0.35 ^a^	27.92 ± 0.89 ^b^	0.57	0.007
NE (ng/L)	88.5 ± 4.01	86.4 ± 6.58	88.89 ± 4.29	101.84 ± 4.68	2.81	0.194
GH (μg/L)	25.61 ± 0.45 ^a^	29.24 ± 1.07 ^b^	26.19 ± 0.51 ^a^	20.7 ± 0.32 ^c^	0.96	<0.001
INS (mIU/L)	42.74 ± 2.31	47.92 ± 2.23	43.89 ± 2.59	40.54 ± 1.78	4.34	0.209

ACTH = Adrenocorticotropic Hormone; ANGII = Angiotensin II; NE = Norepinephrine; GH = Growth hormone; INS = Insulin. Data are presented as mean ± SEM. a–c means within a row with no common superscripts difference (*p* < 0.05).

## Data Availability

The datasets produced and/or analyzed during the current study are available from the corresponding author on reasonable request. The raw data of the 16S rDNA gene sequencing has been shared in the NCBI databases (https://www.ncbi.nlm.nih.gov/sra/PRJNA1280260 (accessed on 6 August 2025)).

## Supplementary Materials

The following supporting information can be downloaded at: https://www.mdpi.com/article/10.3390/ani15172625/s1, Table S1: Elisa kit.

## Abbreviations

ADG, average daily gain; ADFI, average daily food intake; F:G, feed/gain; MDA, Malondialdehyde; T-AOC, total antioxidant capacity; SOD, superoxide dismutase; GSH-px, glutathione peroxidase; GE, Total energy; CP, crude protein; CF, crude fiber; EE, crude fat; DM, dry matter; GLU, Glucose; BUN, urea nitrogen; TG, triglycerides; TP, total protein; TBIL, total bilirubin; ALT, alanine aminotransferase; AST, aspartate amino-transferase; LDH, lactate dehydrogenase; GH, growth hormone; INS, insulin; ANGII, angiotensin II; ATCH, Adrenocorticotropic Hormone; NE, norepinephrine.
