# Supplementary material for: Effects of Different Forage Sources on Growth Performance, Blood Biochemistry, Hormone Concentrations, and Intestinal Microbiota in Alpacas
Source: Animals (Basel). 2025 Sep 8;15(17):2625. doi: 10.3390/ani15172625 (PMC12427470; doi:10.3390/ani15172625)
Supplement: Supplementary file 1 [file animals-15-02625-s001.zip › animals-3767448-supplementary.pdf]

Supplementary Table1. Elisa kit.

Elisa kit	Cat. No	Supplier
GH	JM-00440S2	Jingmei ,Jiangsu, China
INS	JM-07745S1	Jingmei ,Jiangsu, China
ANGII	JM-07998S2	Jingmei ,Jiangsu, China
ACTH	JM-07993S1	Jingmei ,Jiangsu, China
NE	JM-08347B2	Jingmei ,Jiangsu, China
GSH-Px	A005-1-2	Jiancheng ,Nanjing, China
MDA	A003-1-2	Jiancheng ,Nanjing, China
T-SOD	A001-3-2	Jiancheng ,Nanjing, China
T-AOC	A015-2-1	Jiancheng ,Nanjing, China
